# New transformed features generated by deep bottleneck extractor and a GMM–UBM classifier for speaker age and gender classification

**DOI:** 10.1007/s00521-017-2848-4

**Published:** 2017-01-17

**Authors:** Arafat Abu Mallouh, Zakariya Qawaqneh, Buket D. Barkana

**Affiliations:** 1 Computer Science and Engineering Department, University of Bridgeport, Bridgeport, CT 06604 USA; 2 Electrical Engineering Department, University of Bridgeport, Bridgeport, CT 06604 USA

**Keywords:** Speaker recognition, Age and gender, Classification, MFCCs, Deep neural network, DBF extractor

## Abstract

Speaker age and gender classification is one of the most challenging problems in speech signal processing. Recently with developing technologies, identifying speaker age and gender information has become a necessity for speaker verification and identification systems such as identifying suspects in criminal cases, improving human–machine interaction, and adapting music for awaiting people queue. Despite the intensive studies that have been conducted to extract descriptive and distinctive features, the classification accuracies are still not satisfactory. In this work, a model for generating bottleneck features from a deep neural network and a Gaussian Mixture Model–Universal Background Model (GMM–UBM) classifier are proposed for speaker age and gender classification problem. Deep neural network with a bottleneck layer is trained in an unsupervised manner for calculating the initial weights between layers. Then, it is trained and tuned in a supervised manner to generate transformed mel-frequency cepstral coefficients (T-MFCCs). The GMM–UBM is used to build a GMM model for each class, and the models are used to classify speaker age and gender. Age-annotated database of German telephone speech (aGender) is used to evaluate the proposed classification system. The newly generated T-MFCCs have shown potential to achieve significant classification improvements in speaker age and gender classification by using the GMM–UBM classifier. The proposed classification system achieved an overall accuracy of 57.63%. The highest accuracy is calculated as 72.97% for adult female speakers.

## Introduction

This study focuses on generating an efficient and robust feature set by using deep bottleneck feature extractor (DBF) from a DNN and designs a GMM–UBM classifier for speaker age and gender classification. Obtaining the information of age, gender, accent, and emotional state from speech utterance has many applications in language learning, phone ads, criminal cases, computerized health and educational systems, and human–computer interface (HCI). HCI systems can be custom-designed based on speaker’s voice portrait to guide conversations and to improve the level of customer satisfaction [1–4]. All these applications are rapidly emerging and requiring better quality of service. Therefore, more research is needed to improve the performance of current speech signal processing systems to extract speakers’ demographics [5–7].

Speaker age and gender classification is considered as one of the most challenging tasks in the field of speech processing. Challenges in this field include background noise, speech duration, text-dependent or text-independent system design, and accent variance caused by different speakers. Many studies have been carried out on extracting features and designing classifiers for speaker age and gender classification. There are two main stages: (1) feature extraction and (2) classification. Distinctive features from speech signals that represent speaker age and gender information uniquely are calculated in the feature extraction stage. An effective feature set should be unsusceptible to background noise since operating surroundings most likely may have background noise or some type of interference [2]. In addition, an effective feature set should contain age and gender information no matter what the spoken language and utterance. The classification stage centers on designing a classifier that uses the feature set to predict speaker age and gender information.

In general, feature sets can be calculated by using spectral, prosodic, or glottal characteristics of speech utterances. Spectral and temporal features include MFCCs [2, 8–12, 50], formant frequencies, fundamental frequency (F0) [2, 9], energy, relative spectral transform (RASTA) [2], RASTA–perceptual linear prediction (RASTA-PLP) [2], jitter and shimmer [8–10, 13], speech rate [13], harmony, pitch range (PR) [2, 14], and zero-crossing rates. They are used in age and gender identification and classification systems by previous studies. MFCCs are considered to be one of the most effective feature sets in the speech signal processing literature [15]. The main advantage of MFCCs is its ability to model the vocal tract filter in short-time power spectrum. Although MFCCs are currently used for age and, especially, gender classification, the performance of MFCCs is greatly affected by noisy recording environments. This work uses MFCCs as a feature set in order to compare the performance of the proposed transformed MFCCs (T-MFCCs) that are generated based on the original MFCCs and the GMM–UBM classifier.

DNNs have been used for feature extraction and classification in various fields, such as computer vision [16–18], image processing and classification [17, 19], phonetic recognition [20–22], conversational speech recognition [23–26], natural language recognition [27–29], semantic utterance classification [30, 31], hand-writing recognition, audio processing [19, 24], and visual object recognition [32, 33]. The artificial neural network (ANN) is a subfield of artificial intelligence, and it is used as a classifier. The structure of an NN is composed of input layer, one hidden layer, and output layer. On the other hand, a DNN structure is composed of input layer, multiple hidden layers, and output layer. DNN training is performed in two steps. The first step is generative and unsupervised. Each layer is trained separately. The second step is discriminative and supervised.

Ming-li et al. [9] proposed a system which combined five classifiers: GMM-based, GMM with support vector machine (SVM) mean supervector, GMM–SVM maximum likelihood linear regression (MLLR) supervector, GMM–SVM tandem posterior probability (TPP) supervector, and SVM baseline subsystems using 450-dimensional feature vectors including prosodic features. In addition, they combined two or more systems by using fusion technique to increase the classification accuracy. The overall accuracies of the five individual classifiers were 43.1, 42.6, 36.2, 37.8, and 44.6%, respectively. The combined GMM and GMM–SVM mean supervector systems achieved 45.2% of overall accuracy. The fused classifier, the combination of GMM–SVM MLLR supervector and GMM–SVM TPP supervector systems, achieved an overall accuracy of 40.3%. The fusion of the first four classifiers achieved an overall accuracy of 50.4%. Finally, the fusion of the five classifiers performed slightly better by achieving overall accuracy as 52.7%.

Metze et al. [10] studied multiple classifiers for speaker age and gender classification based on telephone applications. They compared the classification results with human performance on the same data. Four automatic classification methods, a parallel phone recognizer (PPR); dynamic Bayesian networks to combine prosodic features; linear prediction analysis (LPA); and GMM based on the MFCCs feature set, were compared. Overall accuracies were reported as 54, 40, 27, and 42%, respectively. Overall classification accuracy of human listeners was reported as 45%.

Myung-Won Lee [11] applied SVM and decision tree (DT) classifiers deploying the MFCCs set in order to build an age and gender classification system for a human robot. They conducted their research on a private corpus. The overall accuracies were reported as 91.39 and 88.37%, respectively, by using MFCCs–SVM and MFCCs-DT for age classification only. The overall accuracies for gender classification by using the same systems were calculated as 93.16, 91.45%. Tobias et al. [34] studied multiple systems with different combinations. A combination of several glottal, spectral, and prosodic feature sets was used in their system. Their system achieved an overall accuracy of 42.2% by the GMM–UBM classifier.

Kim et al. [8] built a home robot that classified speaker age and gender. Three different techniques were used in their work: (1) multilayer perceptron (MLP), (2) GMM, and (3) ANN. Weighted supervised nonnegative matrix factorization (WSNMF) and general regression neural networks (GRNNs) were used by Bahari et al. [12] to design an age and gender regression system. They achieved an accuracy of 96% for gender recognition on a Dutch speech database. For age estimation, the achieved mean absolute error was 7.48 years. Meinedo and Trancoso [35] studied fusion technique by using four classification models. Short- and long-term acoustic and prosodic features were used. The highest classification accuracy was achieved by using linear logistic regression classifier among the four models.

Nisimura and Lee [36] proposed a speech guidance system and used an SVM classifier, which was able to classify adult and children speakers from a private database. Classification accuracy was reported as 92.4% by using acoustic and linguistic features of speech utterances. Dobry et al. [13] proposed a speech dimension reduction method for age–group classification and precise age estimation. After deploying SVM with RBF kernel, they noted that the classifier performance was improved by using their dimension reduction method and the SVM classifier was faster and less affected by over-fitting problem. Muller et al. [37] built a special system for elderly people. Four classes as elderly female, non-elderly female, elderly male, and non-elderly male were studied. Two databases, M31 and ScanSoft, were used to evaluate the system performance by using jitter, shimmer, and speech rate features and *k*-nearest neighbors (KNN), SVM, and naïve Bayes (NB) classifiers.

A brief summary of some of the significant works on speaker age and gender classification is presented in Table 1. Table 2 shows a summary of previous works on age only while Table 3 presents the previous works on gender only.

**Table 1:** Summary of previous works on speaker age and gender classification

Database, #of classes	Features	Classifier	Overall accuracy (%)
aGender, 7 classes [9]	13 MFCCs, 450-dimensional acoustic features, several prosodic features (F0, F0 envelop, jitter, and shimmer)	GMM-based, GMM–SVM mean supervector, GMM–SVM MLLR supervector, GMM–SVM TPP supervector, SVM baseline system, and combined 5 systems	52.7
aGender, 7 classes [10]	MFCCs, several prosodic features (jitter and shimmer), and statistical information	PPR, MLP, dynamic bayesian networks, LPA	45
aGender, 7 classes [34]	Several glottal, spectral, and prosodic	GMM–UBM	42.2
Private, 2 classes (children and adult) [36]	Acoustic and linguistic	MAP	92.4
Private, 4 classes (elderly female, non-elderly female, elderly male, and non-elderly male) [13]	Jitter, shimmer, and speech Rate	KNN, SVM, and NB	–
aGender, 7 classes (child, young female, young male, adult female, adult male, senior female, senior male) [2]	Spectral and temporal features 13 MFCCs, RASTA_PLP, F0, 3PR, 20PR	SVM	63.7%

**Table 2:** Summary of previous works on speaker age classification

Database, # of classes	Features	Classifier	Overall accuracy (%)
Private, 2 classes (children and adults: age ranges are not specified) [11]	MFCCs	SVM and decision tree	91.39
Private, 2 classes (adult and child) [8]	Jitter and shimmer	ANN	96.57
aGender, 4 classes (children, young, adult, and senior) [35]	Acoustic and prosodic	GMM–UBM, MLP, SVM, and linear logistic regression	50.6
aGender, 7 classes (child, young female, young male, adult female, adult male, senior female, senior male) [2]	Spectral and temporal features, 13 MFCCs, RASTA_PLP, F0, 3PR, 20PR	KNN	66.2

**Table 3:** Summary of previous works on speaker gender classification

Database, # of classes	Features	Classifier	Overall accuracy (%)
Private (7 female and 7 male speakers) [11]	MFCCs	SVM and decision tree	93.16
Private [8]	MFCCs	GMM	94.9
Private [12]	MFCCs	WSNMF and GRNNs	96
aGender [35]	Acoustic and prosodic	GMM–UBM, MLP, SVM, and linear logistic regression	86.9
aGender [2]	Spectral and temporal features, 13 MFCCs, RASTA_PLP, F0, 3PR, 20PR	SVM	84.7

## Methodology

**Fig. 1:**
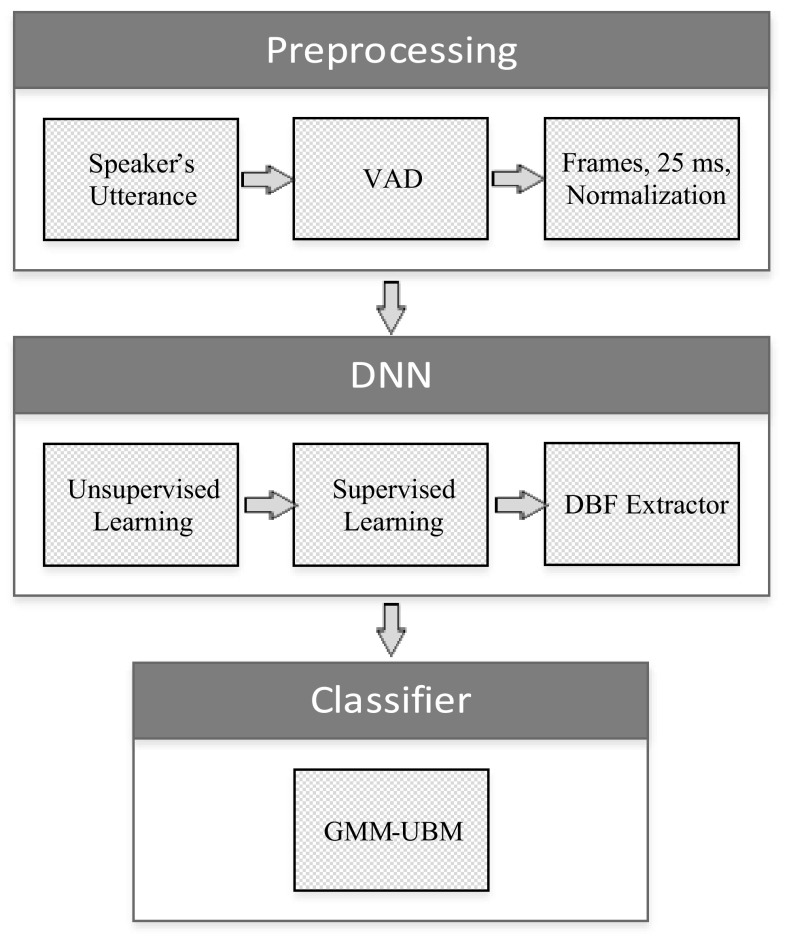
Proposed system

This work proposes a method to generate transformed features and designs a GMM–UBM-based classifier. The proposed system consists of preprocessing, DBF extractor, and GMM–UBM. The block diagram of the system is shown in Fig. 1. Speech utterances are preprocessed, and DNN model is trained by short utterances to generate the transformed features which are fed to the GMM–UBM classifier. The DBF extractor is built by using DNN architecture. First, it is trained in an unsupervised manner for calculating the initial weights between layers. Second, it is trained and tuned in a supervised manner to generate the T-MFCCs. The GMM–UBM is used to build a GMM model for each class; then, the generated models are used to classify each short utterance to a corresponding class.

### Preprocessing

Voice activity detection (VAD) is an essential step in most speech signal processing applications especially if background noise is present. The importance of VAD is due to the fact that it improves the speech intelligibility and recognition. Since the speech utterances in the aGender database were recorded in a public telephone center, the recorded utterances were exposed to noise and other interferences. As a result, VAD algorithm is necessary to reduce background noise and silent epochs in utterances to prepare them for feature extraction. In addition, cepstral mean variance normalization (CMVN) is applied to remove convolutional distortion and the linear channel effects. CMVN can be applied globally or locally. In this work, it is applied globally to get a normal distribution with zero mean and unit variance.

### Pre-training and DNN structure

**Fig. 2:**
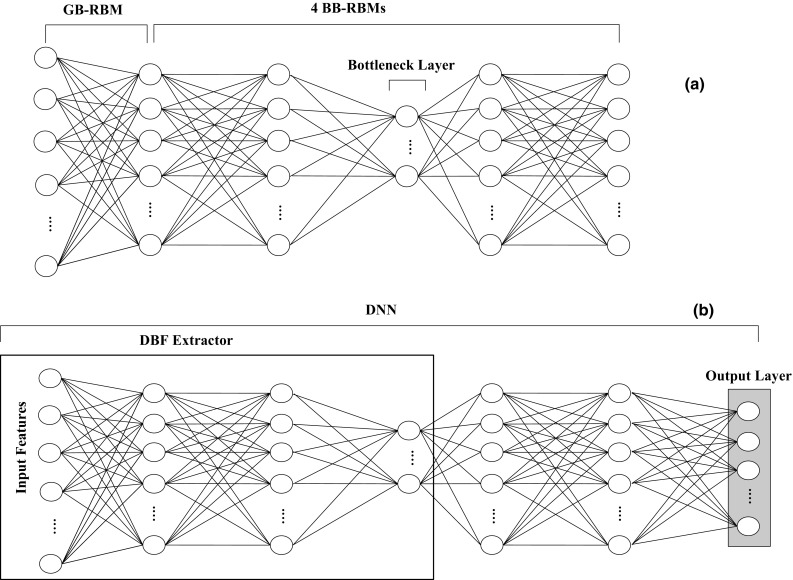
Process of DBF extracting using DNN. **a** Pre-training (unsupervised phase). **b** Fine-tuning (supervised phase)

DNN is considered as one of the most efficient feature extractors and classifiers in machine learning [38]. The training process of DNN is performed in two stages: (1) pre-training and (2) fine tuning. In the pre-training stage, a deep belief network (DBN) architecture which consists of several layers of RBMs is used (Fig. 2a). In here, a bottleneck RBM layer is added into the middle of the DBN architecture, where the number of nodes in this layer is smaller than other layers. The learning of each RBM layer is done independently using an unsupervised learning algorithm. Five RBM layers are used. The first layer is Gaussian–Bernoulli restricted Boltzmann machine (GB-RBM) since the input data are real value. The other four layers are Bernoulli–Bernoulli RBMs (BB-RBMs). After the training is finished, the calculated weights are used to initialize the weights in the supervised phase. Features are extracted by optimizing the parameters of each RBM layer. Each RBM layer is trained separately in a greedy-wise manner. The output of the previous layer is the input to the next RBM. For the BB-RBM, the input layer unit values, *V*, and the output layer unit values, *H,* are binary where *V* and *H* are ϵ{0,1}. The output of each hidden unit is calculated using the energy function as given in (1).

1 $$E\left( {v,h} \right) = - \mathop \sum \limits_{i = 1}^{V} \mathop \sum \limits_{j = 1}^{H} v_{i} h_{j} w_{ij} - \mathop \sum \limits_{i = 1}^{V} v_{i} b_{i}^{v} - \mathop \sum \limits_{j = 1}^{H} h_{j} b_{j}^{h}$$

where *v*_*i*_ is the visible unit in the layer *i* and *h*_*j*_ is the hidden unit in the layer *j*. *w*_*ij*_ denotes the weight between the visible unit *i* and the hidden unit *j*. *b*_*i*_^*v*^ is the bias of the visible unit *i*. *b*_*j*_^*h*^ is the bias of the hidden unit *j*. For the GB-RBM, input layer unit values, *V*, are real numbers where *V* ϵ *R* and the output layer unit values, *H*, are binary where *H* ϵ {0,1}. The output of each hidden unit is calculated by using the energy function as given in (2).

2 $$E\left( {v,h} \right) = - \mathop \sum \limits_{i = 1}^{V} \mathop \sum \limits_{j = 1}^{H} \frac{{v_{i} }}{{\sigma_{i} }}h_{j} w_{ji} + \mathop \sum \limits_{i = 1}^{V} \frac{{\left( {v_{i} - b_{i}^{v} } \right)^{2} }}{{2\sigma_{i}^{2} }} - \mathop \sum \limits_{j = 1}^{H} h_{j} b_{j}^{h}$$

where *σ*_*i*_ is the standard deviation of the Gaussian noise for the visible unit *i*.

RBM is the basic building block in DBN, and it is used as a feature detector. It is trained in an unsupervised manner. A number of RBMs could be stacked and combined together to represent a generative model which is called DBN [23, 39]. There are many advantages of using DBN architecture. It starts by extracting low-level information from the input features and continues refinement until it reaches the upper layers where more compact and abstract explanation of the input features are found. Each RBM tries to extract significant information from the input, but no RBM layer assures to find the best representation of the input features. So, the learning process is done accumulatively across the stack of RBMs until the best representation is reached. The final weights are used to initialize the weights in the fine-tuning phase.

The bottleneck layer architecture with the DBF extraction process is shown in Fig. 2. After the DBN learning is completed and weights are initialized (as shown in Fig. 2a), the output layer of the labels is added on top of the DBN to construct the DNN architecture (Fig. 2b) and the network is re-trained. The bottleneck layer is used to generate the transformed features. These labels are considered to be classes. Then, backpropagation (BP) algorithm is used to tune all the weights on each layer. BP algorithm depends on the difference between the derivatives of the cost function and the final output of the network [40, 41]. In other words, BP algorithm adjusts the network parameters iteratively in a top-to-bottom sequence. By using fine-tuning, the representation of the input features becomes efficient and robust. In this way, we expect to increase the chances for the classifier to get higher accuracy rates than the accuracy rates by using the original input features.

In this work, the output layer of the DNN architecture represents the layer of labels which correspond to the tied-state triphones for all utterances in the database. The tied-state triphone labels are obtained through the forced alignment of trained GMM based on hidden Markov models (HMMs) by using maximum likelihood (ML) and minimum phone error (MPE) techniques.

*DBF Extractor* DBF extractor is generated from the trained DNN. The architecture of the DBF extractor is shown in Fig. 2b. After the DNN training, all layers above the bottleneck layer are removed. With the given DBF extractor with *M* layers, the T-MFCCs features are extracted by using (3).

3 $$\left\{ {\begin{array}{*{20}l} {l_{1} \left( x \right) = \sigma \left( {\mathop \sum \limits_{n = 1}^{N} w\left( {x_{n} + b_{1} } \right)} \right)} \\ { l_{2} \left( x \right) = \sigma \left( {\mathop \sum \limits_{n = 1}^{{F_{2} }} w\left( {x_{n} + b_{2} } \right)} \right)} \\ . \\ . \\ {l_{M} \left( x \right) = \sigma \left( {\mathop \sum \limits_{i = 1}^{{F_{M} }} w\left( {l_{m - 1} \left( x \right) + b_{M} } \right)} \right)} \\ \end{array} } \right.$$

σ is computed by the logistic function $\sigma \left( x \right) = 1/\left( {1 + \exp ( - x)} \right).$ *X* = {*x*_1_, *x*_2_, …, *x*_*N*_} is the feature set vector and *N* is the number of the features in the set. *L*_*M*_ is the output of the layer *M. F* is a varying number that represents the input for each layer in the DBF. *W* represents the weights between the input nodes and output nodes in each layer. The bias for each layer is represented by *b*_*m*_.

The employment of the bottleneck layer has several benefits as eliminating the redundant values from the input feature set by reducing the number of units inside the bottleneck layer and reflecting the class labels during the classification process [42, 43]. Moreover, the bottleneck layer forces the neural layers to filter the input features to keep the descriptive and distinctive features derived from short speech utterances [44].

### GMM–UBM system

GMM is considered to be one of the most effective models that have been used in different fields such as speaker recognition, language identification, and speaker age and gender classification. It is used as a standard classifier for text-independent speaker recognition because of its ability to approximate various arbitrary shaped distributions. One of the most attractive benefits of GMM model is its fast training process compared to other models [45].

**Fig. 3:**
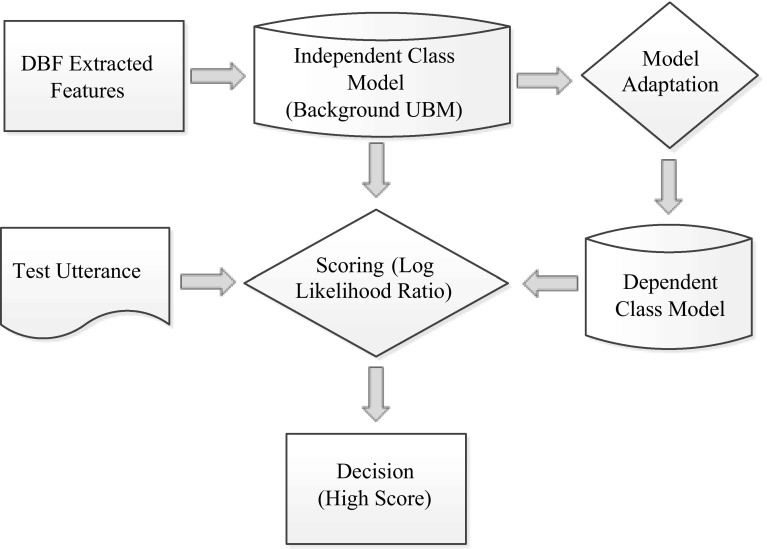
GMM–UBM classifier process

The expectation–maximization (EM) algorithm is used iteratively to create a GMM model for feature vectors that represent a class and to estimate the ML-GMM parameters. The UBM is considered as a well-trained GMM but large enough to model the speaker age and gender distribution of the feature space independently. In other words, the UBM is an independent class of a GMM model that is trained by utterances to reveal speaker’s general characteristics. Mainly the UBM is used as a prior model for the maximum a posteriori (MAP) adaptation model when it tries to estimate class-specific model parameters as shown in Fig. 3. There are two common methods to build a UBM model. The first method creates one UBM that represents all classes together in a database. This method requires well-balanced data between all the classes. The second method creates a UBM for each class in a database; then, all UBM classes are combined into one final UBM model [46].

GMM–UBM system is employed as a classifier in this study. GMM is used to model short utterances. UBM with a MAP model adaptation is used to build the age and gender class models as shown in Fig. 3.

In training, a UBM model is built for all utterances using the T-MFCCs feature set. Each class model is adapted by using the MAP adaptation from the UBM model. In testing, the log likelihood ratio is computed between test utterances, class models, and the UBM model before the final decision is given based on the highest score.

A GMM is a weighted sum of *M* component Gaussian densities. It is given by (4). *M* is the number of Gaussian densities for all vectors, and $w_{i}$ represent the mixture weights. $p_{i} \left( x \right)$, *i* = 1, 2, …, *M*, are the component Gaussian densities. $x$ is a *K*-dimensional feature vector. Gaussian function of the form is given by (5). *μ*_*i*_ are the mean vectors and ∑_*i*_ are covariance matrices. The complete GMM is parameterized by the mean vectors, $\mu_{i} ,$ covariance matrices, ∑_*i*_, and mixture weights, $w_{i} ,$ from all component densities. It is represented by the notation given in (6).

4 $$p\left( {x|\lambda } \right) = \mathop \sum \limits_{i = 1}^{M} w_{i} p_{i} \left( x \right)$$

5 $$p_{i} \left( x \right) = \frac{1}{{\left( {2\pi } \right)^{K/2} \left| {\sum_{i} } \right|^{1/2} }}\exp \left\{ { - \frac{1}{2}\left( {x - \mu_{i} } \right)^{'} \left( {\sum_{i} } \right)^{ - 1} \left( {x - \mu_{i} } \right)} \right\}$$

6 $$\lambda = \left\{ {w_{i} ,\mu_{i} ,\sum i } \right\}\quad {\text{for}}\quad i = 1,2, \ldots ,M$$

In the GMM–UBM approach, each class model is created by combining the class training data and modeling the UBM parameters. There are many ways to adapt the class model from the UBM model. Here, MAP adaptation model is used. The purpose of the MAP model is to optimize the best values of the model parameters which maximize the likelihood of the GMM model for given training utterances. Based on *λ* parameters and vectors of training utterances for each class, MAP adaptation is performed in two steps. (1) The statistics of *λ* parameters of the training utterances are calculated for each mixture in the model. (2) The *λ* parameters of each mixture model are adapted by updating the statistics of the corresponding mixture with the new statistics that are calculated in the first step. It is important to mention that adaptation coefficients are used in the MAP estimation. The adaptation coefficients monitor the updates between the old and new statistics estimation. The values of the adaptation coefficients are usually data dependent for each mixture and *λ*. In practice, it is common to use the same adaptation coefficients for all *λ* parameters. Given a test utterance *X*, *X* = {*x*_*1*_, *x*_*2*_, …, *x*_*M*_}, the log likelihood ratio (llr) is used to compute a score between each class model and the UBM as given in (7). The highest score is taken as the final result.

7 $${\text{llr}}\left( X \right) = \log p\left( {X|\lambda_{{{\text{class }}\;{\text{model}}}} } \right) - \log p\left( {X|\lambda_{\text{UBM}} } \right)$$

## Experimental results and analysis

 In this section, the proposed system is evaluated on the aGender database. Several experiments are conducted to show the effectiveness of the T-MFCCs compared with the original MFCCs.

### Database

aGender corpus is used to test the performance of the proposed T-MFCCs using the GMM–UBM classifier. Each speaker recorded six sessions using a mobile phone, and the sessions were recorded indoor and outdoor to gain diverse environments. The utterances were sampled at 8 kHz and stored in 8-bit with A-Law format. The database consists of 47 h of prompted and free text, which are command words, embedded commands, month, week day, relative time description, public holiday, birth date, time, date, telephone number, postal code, first name, last name, yes/no with according free or preset inventory and according “eliciting” questions as “Please tell us any date, for example the birthday of a family member [47, 48].” The number of speakers in the database is 954, and it includes seven categories of age and gender as shown in Table 4. The number of utterances in the database is 65,364, and the average utterance length is 2.58 s; thus, the utterances are considered as short utterances. The database was divided into two parts; the training set contains 53,076 utterances (770 speakers) while the test set contains 17,332 utterances (25 speakers/class).

**Table 4:** Age-annotated database of german telephone speech database

Class	Category	Age range	Gender	Abb.
1	Children	7–14	Male + female	C
2	Youth	15–24	Female	YF
3	Youth	15–24	Male	YM
4	Adult	25–54	Female	AF
5	Adult	25–54	Male	AM
6	Senior	55–80	Female	SF
7	Senior	55–80	Male	SM

### MFCCs

**Fig. 4:**
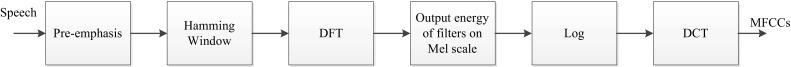
Mel-frequency cepstral coefficients

MFCCs set is one of the most well-known spectral feature sets and has been widely used in many speech applications. In this work, MFCCs are employed. Figure 4 represents the flow diagram of MFCCs calculation.

The window size is chosen as 25 ms which is in the range of 20–40 ms per frame. This window duration is chosen to ensure the quasi-stationarity of the speech signal. Window size has a considerable effect on cepstral coefficients. If the window size is less than two pitch periods long, the cepstral coefficients may not show periodicity in the spectrum. At least two clearly defined periods should remain in the windowed speech segment [49]. In nature, the properties of speech signals change rapidly over time. Discrete Fourier transform (DFT) is used to calculate the power spectrum of each frame. A narrow mel-frequency filter bank is used for low frequencies while a wide mel-frequency filter bank is used for high frequencies. The main point of using the mel-frequency filter bank is to determine the energy level of different frequency ranges. In order to model the human ear, the log is taken for the filter bank energies. The discrete cosine transform (DCT) of the outputs of the log filter bank is calculated. In this work, speech utterances are divided into frames with 25-ms window size. 12 MFCCs and a normalized energy with their first and second derivatives (∆’s and ∆∆’s) are calculated for each frame, resulting in 39 coefficients representing each frame.

### DNN settings

Tied-state triphones which are constructed using a GMM–HMMs-based phone recognizer are trained with ML. The GMM–HMMs of our database have 4400 tied-state triphones, each of which is a GMM composed of 256 Gaussian components.

The DNN settings in our work are based on the work of automatic speech recognition (ASR) in [25, 42]. The DNN configuration has 5 hidden layers where the number of nodes in each layer is 2048 except the bottleneck layer. The bottleneck layer had 39 nodes. The number of nodes in the input layer represents *n* × 39 features, where the target frame is concatenated with its neighboring (*n* − 1)/2 preceding frames and its neighboring (*n* − 1)/2 following frames. We used 11 concatenated frames where the target frame is concatenated with its neighboring 10 frames as five frames before it and five frames after it (5 + 1 + 5). The number of bottleneck layer units is 39. The number of nodes in the output layer is set to 4400 states which represent the number of tied-state triphones in the database.

The training data are divided into minibatches. Each minibatch consists of 1024 random short utterances. In the unsupervised phase, 20 epochs are used to train the GB-RBM. 10 epochs are used for the BB-RBMs. Gibbs sampling and contrastive divergence algorithm are performed on each RBM. A small value for the learning rate in both GB-RBM and BB-RBM is used (0.0025). The number of epochs is 12 in the fine tuning phase. The learning rate is initially set to 0.1 for the first 6 epochs, and then, it is decreased to half of its previous value for the remainder.

### Classification accuracies

The DNN architecture is used to generate T-MFCCs feature set based on the original MFCCs set. Table 5 shows the classification accuracies of the original and T-MFCCs sets for seven age and gender classes. By using the proposed GMM–UBM classifier, T-MFCCs achieved classification accuracies up to 72.97% while the original MFCCs achieved up to 63.78% classification accuracy.

**Table 5:** Overall classification accuracies of the original and T-MFCCs (%)

	C1(C)	C2(YF)	C3(YM)	C4(AF)	C5(AM)	C6(SF)	C7(SM)	Overall Acc
Original MFCCs	63.78	59.46	44.32	24.32	29.19	45.41	38.38	43.55
T-MFCCs	67.02	62.16	41.62	72.97	34.81	52.97	71.89	57.63

 The overall accuracy for the T-MFCCs set is calculated as 57.63% while it is 43.55% for the original MFCCs. The system achieved a significant improvement especially for the adult female class (72.97%) and senior male class (71.89%), whereas accuracies are calculated to be 24.32 and 38.38%, respectively, by using original MFCCs set. The confusion matrices for both feature sets by the GMM–UBM classifier are presented in Tables 6 and 7.

**Table 6:** Confusion matrix for seven age and gender classes by original MFCCs set (%)

	C1(C)	C2(YF)	C3(YM)	C4(AF)	C5(AM)	C6(SF)	C7(SM)
C1 (C)	**63.78**	20	0	3.24	0.54	11.35	1.08
C2 (YF)	22.70	**59.46**	9.19	6.49	0	2.16	0
C3 (YM)	8.65	0	**44.32**	3.24	31.35	5.41	7.03
C4 (AF)	21.08	25.95	2.70	**24.32**	3.24	22.16	0.54
C5 (AM)	3.24	0.54	27.03	17.30	**29.19**	0	22.70
C6 (SF)	8.65	5.95	20.54	7.03	3.78	**45.41**	8.65
C7 (SM)	0.54	0	21.62	5.41	32.43	1.62	**38.38**

Bold values indicate the percent of samples correctly classified

**Table 7:** Confusion matrix for seven age and gender classes by of T-MFCCs set (%)

	C1(C)	C2(YF)	C3(YM)	C4(AF)	C5(AM)	C6(SF)	C7(SM)
C1 (C)	**67.03**	17.30	0	3.24	0	11.89	0.54
C2 (YF)	16.75	**62.16**	10.30	9.19	0	1.62	0
C3 (YM)	7.57	0	**41.62**	2.70	32.97	7.03	8.11
C4 (AF)	1.62	5.40	2.16	**72.97**	2.16	15.70	0
C5 (AM)	3.24	0.54	21.95	17.30	**34.81**	1.08	21.08
C6 (SF)	8.64	4.65	15.13	5.40	4.32	**52.97**	8.65
C7 (SM)	2.16	0.54	7.56	0.54	15.13	2.16	**71.89**

Bold values indicate the percent of samples correctly classified

It is observed that the children, senior male, and adult female classes achieved better results than the other classes. The major confusion occurred between the same genders except for the children and young female classes. This may be due to the fact that the children class contains both male and female speakers who are between 8 and 14 years old. The misclassification between classes may be due to the nature of the MFCCs feature set. The statistical analysis of the MFCCs for seven age and gender classes is studied by Barkana and Zhou [2]. They observed that the MFCCs of all seven classes have near-identical symmetrical distribution and same flatness with minor differences. As a result, they stated that MFCCs set is not adequate to represent each age and gender class uniquely. Although MFCCs are not sufficient to classify age and gender distinctively, the proposed T-MFCCs feature set is proved to be defining the seven age and gender classes more distinctively compared to the original MFCCs.

**Fig. 5:**
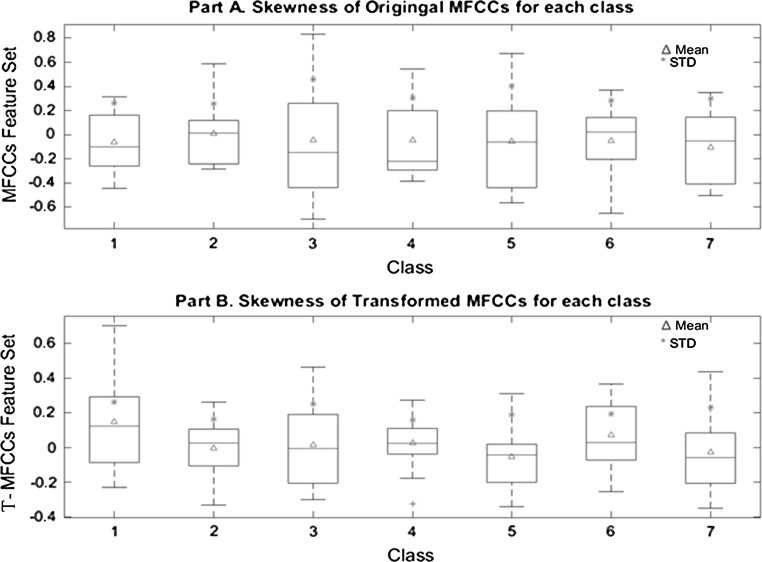
Distributions of original and T-MFCCs feature sets

Statistical analysis of the original MFCCs and T-MFCCs has been conducted for the seven classes to understand the differences further. Figure 5a, b shows the skewness distribution for both sets. Figure 5a shows that the distribution of original MFCCs is skewed to the right for classes C3 (YM) and C4 (AF) while it is skewed to the left for classes C6 (SF), C7 (SM). Classes C1 (C), C2 (YF), and C5 (AM) have nearly symmetrical distribution. Lower and upper quartiles of male speakers (C3, C5, and C7) are observed to be very close. In Fig. 5b, the T-MFCCs set has presented a different distribution than that of the original MFCCs. All classes have nearly symmetrical distribution of the features as the mean and median are the same. However, lower quartile and upper quartile for each class are different. Also, it is clear from Fig. 5 that the T-MFCCs set has completely different features than the original MFCCs set to describe each class.

**Fig. 6:**
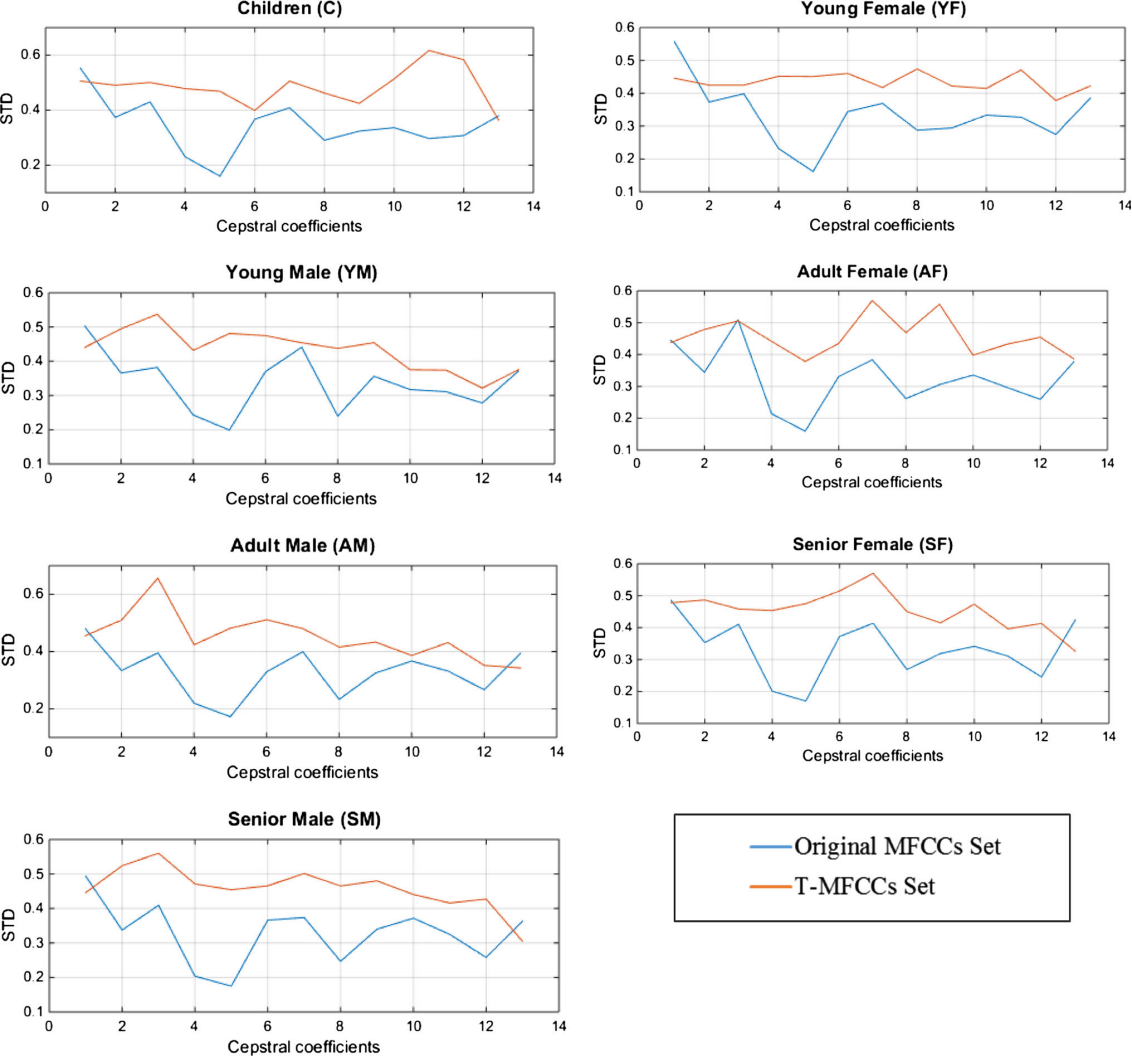
Variation between standard deviation values of the first 13 coefficients of the original and T-MFCCs sets for all classes

Another analysis to compare the T-MFCCs and the original MFCCs is done by comparing variations between standard deviation of the 12 MFCCs and a normalized energy parameter for each class. It is shown in Fig. 6. It is observed that T-MFCCs present less intraclass variation than the original MFCCs. It is also observed that there is significant interclass variation in the T-MFCCs. Low intraclass variation and high interclass variation in features are preferred in order to have better classification.

The improvement in the classification of the adult male class is not significant. The speakers in this class are misclassified as young male or senior male. The statistical analysis of the original MFCCs and T-MFCCs shows that the distribution of the first thirteen cepstral coefficients among the male classes is similar in terms of standard deviation (Fig. 6). Although T-MFCCs provide more uniform intraclass presentation than the original MFCCs, both have similar interclass distribution for male classes. We believe that is why no significant improvement observed in age and gender classification of male speakers.

**Fig. 7:**
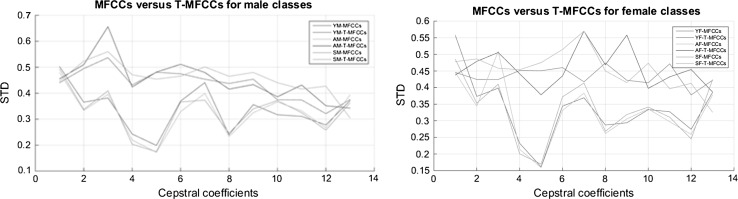
MFCCs versus T-MFCCs sets for all male and female classes

The adult classes represent a wide range of ages between 25 and 54 years old for female and male speakers. While the lower end of the adult classes is close to youth classes, the upper end is close to senior classes. The T-MFCCs features have good variance among female classes (Fig. 7). It is reflected as an increase in the classification accuracies for female speakers. On the other hand, from (Fig. 7), it is observed that the MFCCs and T-MFCCs features have less variance among the male classes compared to that of female classes. As a result, misclassification occurred among male classes, especially between young- and adult male classes. The adult male class can be split into two subclasses as adult male_1 (25–40 years old) and adult male_2 (41–54 years old) in order to increase the classification accuracies. This approach may increase the variance in the T-MFCCs features between the male classes. As a result, the classification accuracies of the adult male classes might be increased. The aGender database has been prepared by Schuller et al. [47] with seven classes. The specific ages of the subjects in each class are not provided in the database. Therefore, we were not able to split the adult male class into two subclasses to evaluate the performance of our system in such a scenario.

In order to assess the number of hidden mixtures effect in a GMM system, several experiments are conducted with different number of hidden mixtures. The results are shown in Table 8. It is noticed that the increase in the number of hidden mixtures up to 128 for both feature sets improved the overall classification accuracy, which stayed nearly unchanged between 128 and 1024 hidden mixtures. The overall accuracy of the system started to decline after the number of mixtures is 1024.

**Table 8:** Overall classification accuracy versus the number of hidden mixtures (%)

	Number of hidden mixtures						
	32	64	128	256	512	1024	2048
Original MFCCs	42.93	43.86	44.48	43.55	43.55	43.63	42.85
T-MFCCs	54.52	55.44	57.07	57.07	56.83	57.99	57.29

The best overall accuracy for the T-MFCCs is observed at 1024 mixtures while it is 128 mixtures for the original MFCCs. The selection of the number of mixtures is problem specific. It is usually chosen by conducting several experiments to find the optimal number of hidden mixtures.

## Conclusions

In this paper, we have proposed a new feature set, T-MFCCs, generated by using a DBF extractor from a DNN. A GMM–UBM classifier is designed to classify speaker age and gender. The aGender corpus is used to evaluate the proposed work.

Optimization of DNN parameters, such as number of layers, number of units in each layer, weight initialization, and learning rate, is problem dependent. The settings of the optimal parameters differ from one problem to another. Therefore, different experiments are conducted to find the optimal settings for the DNN. Optimizing one parameter alone does not optimize the rest of the parameters. Thus, the parameters should be tuned together to reach the optimal settings. To evaluate the performance of the proposed system, we have conducted several experiments with different numbers of hidden mixtures in the GMM–UBM system. Experimental results showed the effectiveness of the T-MFCCs set by significant improvements in classification accuracies. The proposed T-MFCCs set and classification system achieved classification accuracies of 67.02, 62.16, 41.62, 72.97, 34.81, 52.97, and 71.89% for children, young female, young male, adult female, adult male, senior female, and senior male, respectively. The overall accuracy of the proposed system is calculated as 57.63%.

The experimental results revealed that the T-MFCCs set showed better performance than the original MFCCs set especially for female speakers. We did not observe significant performance differences for young and adult male classes by using both feature sets. Based on the statistical analysis of the first thirteen T-MFCCs and the original MFCCs coefficients, the male speakers have similar spectral distribution for both feature sets. The adult classes have a wide age range from 25 years old to 54 years old. Adult classes could be split into two subclasses to improve the classification accuracies of the young and adult male speaker classes. However, it was not possible to investigate this idea by using the aGender corpus since it did not contain the specific age information of each speaker in the database.

As future work, the proposed system can be combined with other spectral and morphological features to improve the classification accuracies. Furthermore, different databases can be used to demonstrate the effectiveness of the proposed system. The effects of the window size on feature sets and classification performance can also be explored as future work.
